# Pneumothorax as a rare complication of peripherally inserted central catheter (PICC) in neonates: A case report study

**DOI:** 10.1016/j.ijscr.2021.106472

**Published:** 2021-10-07

**Authors:** Rasoul Goli, Sina Zafarmokhtarian, Mahmoodreza Ghalandari, Navid Babakeshi-sheytanabad, Sajjad Rostami, Hossna Farajollahi

**Affiliations:** a Department of Medical-Surgical Nursing, School of Nursing and Midwifery, Urmia University of Medical Sciences, Urmia, Iran; b Department of Nursing, School of Nursing and Midwifery, Islamic Azad university of Marand, East Azerbaijan, Iran; c Department of Nursing, School of Nursing and Midwifery, Urmia University of Medical Sciences, Urmia, Iran; d Department of Nursing, School of Nursing and Midwifery, Islamic Azad university of Zanjan, Zanjan, Iran

**Keywords:** Pneumothorax, Catheterization, PICC placement, Neonate

## Abstract

**Introduction and importance:**

The Peripherally Inserted Central Catheter (PICC) placement is associated with complications such as deep vein thrombosis, phlebitis, air embolism, infection, and superior vena cava syndrome. The aim of this study is to report pneumothorax as a rare complication of PICC insertion in a newborn.

**Case presentation:**

The present case report is of a 32-week- and 4-day-old female fetus who was born at Mahzad Obstetrics and Gynecology Hospital, Urmia, Iran. A PICC was placed for the infant. The infant underwent an antero–posterior chest X-ray, in which the presence of a complete white-out of the right hemithorax indicated pneumothorax and right lung collapse. The review of literature in this field showed that there were no reports of pneumothorax as a complication of PICC insertion in neonates.

**Clinical discussion:**

Despite that the PICC placement seems to have many medical advantages in infants, it may cause life-threatening complications such as pneumothorax. In this newborn, the PICC placement was the main cause of pneumothorax and it can be stated that the catheter tip might cause trauma to the chest wall during the placement procedure which resulted in an air trap in the pleural cavity and eventually right lung collapse.

**Conclusion:**

There are a couple of rare cases being reported to have complications of PICC placement in neonates, but none had associated pneumothorax and PICC placement in neonates. Therefore, innovative methods require to be used for meeting the nutrition and fluid requirements of the infants for a long time.

## Highlights

• PICC placement is associated with life-threatening complications.
• Pneumothorax is a rare complication of PICC placement in neonates.
• Innovative methods are required for neonate’s nutrition requirements for a long time.

## 1 Introduction

In recent years, despite many advances in pregnancy care, the overall prevalence of preterm births has increased so that higher numbers of premature infants are hospitalized to receive necessary fluids and electrolytes, intravenous (IV) nutrition, and medications [1]. Therefore, having access to a venous line is one of the common problems in Neonatal Intensive Care Units [2]. Despite the tendency to start oral feeding early and cut off the parenteral nutrition, many of these infants still need a safe central IV line for a long time [3].

Frequent attempts to access a peripheral venous line using angiocatheters expose the infant to further injury since it impairs the health of the infant's highly sensitive skin, which is considered as the first line of defense against systemic infections [4]. Concerning the long-term hospitalization of premature infants in the Intensive Care Units (ICUs) and the lower stability of the peripheral venous lines, it is necessary to find another way of having venous access [5].

Since 1970, the Central Venous Catheters (CVCs) have become the main way of meeting the fluid and nutritional requirements of infants who are not ready to receive oral nutrition [1]. The placement of CVC is conducted under general anesthesia in the operating room using standard surgical methods so that it requires special techniques and is associated with serious complications, such as pneumothorax, hemothorax, and bleeding. In addition, this procedure requires experienced staff and is economically expensive [6]. All of the above reasons led to the invention of a new method of venous access in 1980 called a Peripherally Inserted Central Catheter (PICC) [5], [6].

The PICC facilitated central venous access since it is placed without the need for anesthesia and can be used for weeks with minimal complications [7]. The PICC is a thin, long catheter (14-16 in.) made of soft, flexible silicone or polyurethane [8]. This tube is usually inserted in large peripheral veins (to give IV medications over a long period of time because a large vein can tolerate an IV catheter for a longer time than a small vein) of the arm such as cephalic or basilic vein near the elbow and directed to the Superior Vena Cava (SVC) [9]. Regarding the long-term longevity of PICC, it prevents frequent IV insertions for the infant [8].

Despite the medical advantages of PICC, this method can cause complications such as pain, nerve or tendon damage, Deep Vein Thrombosis (DVT), phlebitis, air embolism, infection, and Superior Vena Cava Syndrome (SVCS) [10]. The review of literature in this field showed that there were no reports of pneumothorax as a complication of PICC insertion in neonates. The aim of this study is to report pneumothorax as a rare complication of PICC insertion in a newborn. This case report was reported according to the SCARE 2020 Guidelines to ensure the quality of reporting [11].

## 2 Case presentation

The present case report is of a 32-week- and 4-day-old female fetus through cesarean section at Mahzad Obstetrics and Gynecology Hospital, Urmia, Iran. The mother had a history of miscarriage and a normal delivery at the age of 32. She was also from a family with a good socioeconomic status resided in Urmia city. During this pregnancy, she had regularly visited a gynecologist for receiving health examinations, in which the fetus was shown to be completely healthy on a color Doppler ultrasound. The mother did not mention any particular drug history and also denied a history of substance or alcohol use. However, she mentioned a history of smoking (one cigarette a day for 10 years).

**Fig. 1:**
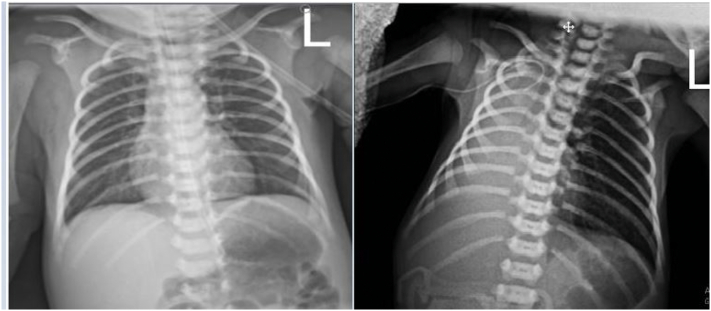
Radiograph of the newborn baby's chest before (left) and after (right) the PICC placement.

**Fig. 2:**
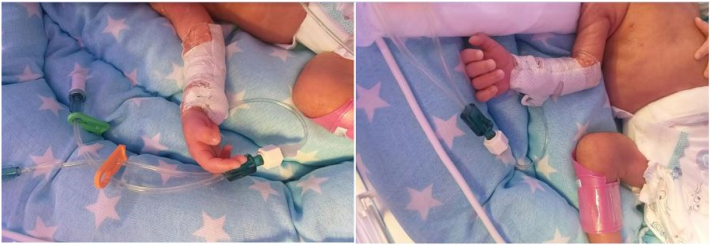
The PICC placement into the basilic vein.

After the onset of labor signs and contractions, the mother went to the hospital and gave birth to a 32-week-and 4-day-old female fetus with a birth weight of 1800 g, height of 42 cm, and head circumference of 32 cm. Vital signs of the infant at birth were as follows: Temperature: 36.7, Heart Rate: 163 bpm, Respiration Rate: 66 bpm, Blood Pressure: 63/24 mmHg, Oxygen Saturation: 98%. The infant's Apgar score was 7 and 8 at birth and 5 min after birth, respectively. Concerning the preterm birth and respiratory distress, the infant was transferred to the NICU and placed in an incubator at an appropriate temperature. On the first day after birth, the infant underwent a chest radiograph, the results of which showed no health problem (Fig. 1). After sterile pre-procedural prepping and draping, a trained nurse first inserted the PICC into the basilic vein using a special angiocatheter and directed it into the vein using an angiocatheter guide (Fig. 2). Then the catheter (the Arrowg+ard Blue Advance™ PICC made in United States country), was pushed from the catheter insertion site to the middle of the clavicle bone and then to the mid-sternal region. Finally, the catheter was fixed with an IV fixing bandage (sterile disposable cannula catheter fixing IV Non-woven dressing made in China). After the PICC placement, the infant underwent an anterior-posterior chest X-ray again and a neonatologist confirmed the correct placement of the PICC tip in the SVC. In addition, the catheter insertion site was dressed with a sterile transparent dressing based on the medical protocol to make the catheter insertion site visible and timely diagnose local complications. Two hours after the PICC placement, the infant's oxygen saturation decreased and her vital signs changed as follows: Temperature: 36.9, Heart Rate: 199 bpm, Respiration Rate: 76 bpm, Blood Pressure: 60/25 mmHg, Oxygen Saturation: 45%. Despite 8-liter-per-minute oxygen therapy using an oxygen tent, oxygen saturation did not increase so that the patient received Continuous Positive Airway Pressure (CPAP) ventilation with the Fraction of Inspired Oxygen (FiO_2_) = 80% and the Positive End-Expiratory Pressure = 5 cm H_2_O. The infant underwent a chest radiograph again, in which the presence of a complete white-out of the right hemithorax indicated pneumothorax and right lung collapse (Fig. 1). Moreover, the reduction of right lung sounds indicated a collapse of the right lung, because of which a chest tube (disposable medical closed chest drainage bottle made in china) was immediately placed in this region.

## 3 Discussion

Venous access is an important dimension of treatment in neonates [3]. The use of CVCs, the placement of which is conducted using peripheral veins, has been considered as an important part of treatment procedures among neonates in recent years [1]. It is estimated that about 8.3-33% of infants admitted to the Neonatal Intensive Care Units will need the PICC placement [12]. The PICC has many medical advantages such as ease of use, cost-effectiveness, and safe placement in the body [13]. Despite that the PICC is often used for premature infants, it can cause some complications, especially infections [10]. Catheter-Related Bloodstream Infections (CRBSIs) account for about 70% of nosocomial infections in the Neonatal Intensive Care Units [14].

Other common complications of PICC placement include blockage, leakage, thrombosis, and pneumothorax [10]. However, pneumothorax rarely occurs in infants with the PICC line [15]. Pneumothorax refers to the presence of air in the pleural cavity that may occur spontaneously or as a result of trauma [16]. A spontaneous pneumothorax occurs without a history of chest trauma and has two types including primary (without underlying lung disease) or secondary (due to underlying lung disease) [17]. Traumatic pneumothorax is caused by penetrating or non-penetrating blows to the chest [16]. A tension pneumothorax refers to a type of pneumothorax in which the pressure inside the pleural cavity remains positive throughout the respiratory cycle [17]. This type of pneumothorax usually occurs during mechanical ventilation or cardiopulmonary resuscitation [18]. Positive pleural pressure is a life-threatening condition since it causes severe ventilatory impairment so that the positive pressure is transmitted to the mediastinum, resulting in reduced venous return and decreased cardiac output [16]. Pneumothorax may occur during lung biopsy or pleural drainage due to the perforation of the visceral pleura and the leakage of air into the lungs [15].

In this patient, the PICC placement was the leading cause of pneumothorax and it can be stated that the catheter tip might cause trauma to the chest wall during the placement procedure which resulted in an air trap in the pleural cavity and eventually right lung collapse. Treatment for spontaneous pneumothorax includes fine-needle aspiration, tube thoracostomy, thoracotomy, and thoracoscopic surgery, resection of the lesion, and stitching the leakage site. In cases where the leakage site cannot be identified, a pleural aberration is used to stimulate adhesion between the parietal and the visceral pleura [18]. In complicated cases, pleurectomy is performed with the separation of the entire parietal pleura from the lower ribs and intercostal muscles [19].

PICCs have the benefit of being inserted at the bedside without general anesthesia and remaining *in situ* for days or weeks with minimal mechanical complications [10]. Regardless of where and how they are inserted, dangerous complications have been associated with the use of central catheters. In contrast to central catheters, PICC are less invasive and also in-expensive [15].

Jain et al. showed that most of neonates with PICC placement had positive blood cultures giving an overall incidence of catheter-associated blood stream infections (CABSI) as 13.4% [20]. Harfi et al. reported a preterm baby, with low birth weight who developed large pericardial effusion as a complication of total parenteral nutrition *via* a peripherally inserted central catheter, managed successfully with pericardiocentesis [21]. Moreover, Sertic et al. showed among 3454 neonates with PICCs, 15 cases had perforation, which resulted in devastating complications such as pericardial and pleural effusions [8]. The review of literature in this area indicated that there were no reports of pneumothorax as a complication of PICC placement in neonates.

## 4 Conclusion

There are a couple of rare cases being reported to have complications of PICC placement in neonates, but none had associated pneumothorax and PICC placement in neonates. Potential injury to the lung parenchyma by the tip of the PICC catheter resulted in further development of a pneumomediastinum. Therefore, in line with the advancement of science and technology in the modern world, alternative as well innovative methods such as selecting alternative pathway/over the wire (SAP/OTW), percutaneous transluminal angioplasty (PTA), re-puncture in ipsilateral arm (RIA), and catheter placement in the contralateral arm (CICA) [22] require to be used for meeting the nutrition and fluid requirements of the infants who may be admitted to the Neonatal Intensive Care Units for a long time.

## Provenance and peer review

Not commissioned, externally peer-reviewed.

## Sources of funding

This case report did not receive any specific grant from funding agencies in the public, commercial, or not-for-profit sectors.

## Ethical approval

All ethical principles were considered in conducting this case report. All patient information kept confidential.

## Consent

Written informed consent was obtained from the patient for publication of this case report and accompanying images. A copy of the written consent is available for review by the Editor-in-Chief of this journal on request.

## Authors' contributions

Rasoul Goli, Sina Zafarmokhtarian, and Mahmoodreza Ghalandari: Study concept, data collection, writing the paper and making the revision of the manuscript following the reviewer's instructions. Navid Babakeshi-sheytanabad, Sajjad Rostami, and Hossna Farajollahi: Study concept, reviewing and validating the manuscript's credibility. Rasoul Goli: reviewing and validating the manuscript's credibility.

## Research registration

Not applicable.

## Guarantor

Rasoul Goli.

## Declaration of competing interest

None.
